# Carcinoma ex pleomorphic adenoma containing two cancerous components: two case reports

**DOI:** 10.3389/fonc.2026.1981963

**Published:** 2026-10-22

**Authors:** Qiuyue Mou, Dan Li, Dan Qin, Kaiyu Li, Minhai Nie, Yaoqiang Liu, Xuqian Liu

**Affiliations:** 1 Department of Basic Medical Sciences of Oral Medicine, Affiliated Stomatological Hospital of Southwest Medical University, Luzhou, Sichuan, China; 2 Key Laboratory of Oral and Maxillofacial Prosthetic Reconstruction and Regeneration Luzhou City, Luzhou, Sichuan, China; 3 Institute of Stomatology, Southwest Medical University, Luzhou, Sichuan, China; 4 Department of Pathology, the Second Hospital of Hebei Medical University, Shijiazhuang, Hebei, China; 5 Department of Oral and Maxillofacial Surgery, the Second Hospital of Hebei Medical University, Shijiazhuang, Hebei, China

**Keywords:** carcinoma ex pleomorphic adenoma, mucoepidermoid carcinoma, adenoid cystic carcinoma, salivary duct carcinoma, case report

## Abstract

Carcinoma ex pleomorphic adenoma (CXPA) is a rare epithelial malignancy arising from benign pleomorphic adenoma. Cases with two distinct malignant components are exceedingly rare. Nonspecific clinical and imaging features and heterogeneous histology hinder differentiation from other salivary gland tumors, risking misdiagnosis and suboptimal therapy. We report two CXPA cases with dual malignant components and review the literature. Diagnosis relied on histopathology, immunohistochemistry, special stains, and fluorescence *in situ* hybridization (FISH); both cases showed two malignant components. Histopathology with IHC and FISH was essential, revealing morphological diversity and diagnostic complexity. Literature review confirms rarity and the inadequacy of preoperative clinical and radiological evaluation alone for distinction. Dual-malignant CXPA is a rare diagnostic challenge. Our observations may aid clinicians in reducing misdiagnosis and guiding management.

## 1 Introduction

Pleomorphic adenoma (PA) is the most common salivary gland tumor, accounting for over 50% of epithelial salivary gland neoplasms. According to WHO classification, malignant transformation of PA includes CXPA, carcinosarcoma, and metastatic PA (1). CXPA, defined as epithelial malignancy in benign PA, is predominant (2). CXPA is commonly seen in middle-aged and elderly patients and often arises from a long-standing PA. Patients usually have a history of an untreated or recurrent PA (3, 4). The malignant component in CXPA is heterogeneous, with salivary duct carcinoma and adenocarcinoma, not otherwise specified (NOS), being the most common. Other types such as mucoepidermoid carcinoma, adenoid cystic carcinoma, myoepithelial carcinoma, and adenosquamous carcinoma are less frequent. Cases are usually dominated by a single malignant component, while the coexistence of multiple distinct malignant components is rare. There have been reports of CXPA containing both squamous cell carcinoma and ductal carcinoma (5–7), as well as cases with myoepithelial carcinoma, ductal carcinoma, and adenocarcinoma NOS (8). However, cases with both mucoepidermoid and adenoid cystic carcinoma, or both mucoepidermoid and salivary duct carcinoma, have not been reported. We present two CXPA cases with clinical, histopathological, immunohistochemical, special staining, and FISH data, to aid clinicians in reducing misdiagnosis and improper treatment.

## 2 Case presentation

### 2.1 Case 1

A 74-year-old female presented with a 4-year history of an untreated palatal mass that had painlessly enlarged over the past six months, no previous intervention measures. Physical examination revealed a firm, poorly-defined, non-tender mass (approximately 2.5×2.5×2 cm) with a smooth surface on the palate. Clinical examination and intraoral photography demonstrated a localized palatal swelling covered by intact, smooth mucosa, with mild erythematous discoloration and no apparent surface ulceration (**Figure 1(i)**). Non-contrast CT scan showed an irregular palatal mass (**Figure 1(ii)**). Her medical history included a 3-year history of hypertension and a 6-year history of diabetes mellitus. No significant family history or specific allergies were noted. Detailed psychosocial and genetic history was not available in the archival medical records.

**Figure 1:**
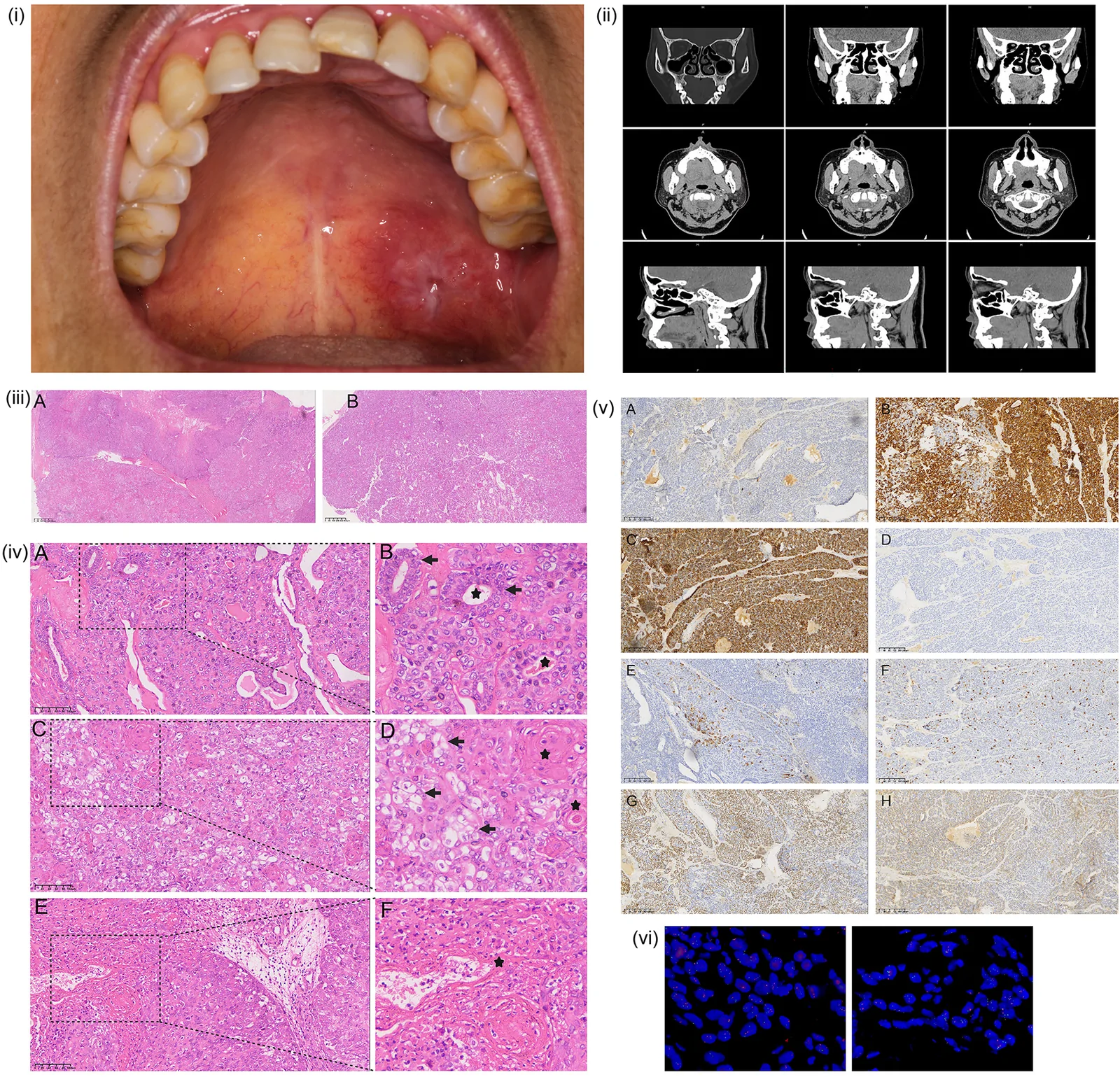
Clinical, radiological, histopathological, immunohistochemical, and molecular findings of case 1. **(i)** Clinical photograph showing a localized palatal swelling covered by intact mucosa, with mild erythematous discoloration and no apparent ulceration. **(ii)** Coronal, axial, and sagittal images of the radiographic findings in Case 1. **(iii)** Representative low-power H&E images showing marked histological heterogeneity and the coexistence of morphologically distinct areas within the tumor **(A, B)**. **(iv)** Histopathological findings of Case 1. **(A, B)** Bilayered glandular structures (black arrows) containing homogeneous eosinophilic mucus (★) within their lumina. Myoepithelial components and myxoid stroma are also evident. **(C, D)** Mucous cells (black arrows) and solid epithelial nests (★) are present. **(E, F)** Tumor cell nests are visible, with larger nests showing central necrosis (★). **(v)** Immunohistochemical results of Case 1. **(A)** CD10(-), **(B)** CK34βE12(+++), **(C)** CK5/6(+++), **(D)** Desmin(-), **(E)** GFAP(+), **(F)** Ki-67(1-2%+), **(G)** p63(+++), **(H)** SMA(+). **(vi)** FISH results of Case 1. Using break-apart probes targeting the *PLAG1* gene locus, the detection of split red and green signals indicates a rearrangement of the *PLAG1* gene. The fused yellow signals represent an intact gene locus.

#### 2.1.1 Preoperative diagnosis: palatal mass, nature to be determined

Operative Findings: On October 8, 2020, the patient underwent excision of the palatal mass. Intraoperatively, a firm, poorly demarcated mass measuring 2.0 × 2.0 × 1.5 cm was identified. The resected specimen was properly collected and submitted for pathological examination.

Pathological Examination: Microscopic examination revealed a tumor with diverse cellular morphology and complex architecture. Representative low-power H&E sections demonstrated marked histological heterogeneity, with morphologically distinct areas distributed throughout the tumor and an overall heterogeneous growth pattern (**Figures 1(iii)A, B**). Within the tumor tissue, glandular epithelial cells displayed cubic to low columnar shapes, with round or oval nuclei exhibiting a vacuolated appearance. Homogeneous eosinophilic mucus was observed within the lumina of gland-like structures, and myxoid tissue was distributed throughout the tumor tissue (**Figures 1(iv)A, B**). The tumor also showed sheet-like distributions of mucoid cells and epidermoid cells, along with solid epithelial nests (**Figures 1(iv)C, D**). Some cells were arranged into epithelial masses, with larger masses exhibiting central necrosis and visible mitotic figures (**Figures 1(iv)E, F**).

Additional pathological parameters: The intraoperative tumor size was approximately 2.0 × 2.0 × 1.5 cm. Focal central necrosis and mitotic figures were identified, although no quantitative mitotic count was available. Detailed histological margin status, lymphovascular invasion, depth/extent of invasion, overall histological grade, and complete pathological stage were not reliably documented in the archived records. No neck dissection was performed; therefore, pathological nodal status and extranodal extension could not be assessed.

Immunohistochemical findings: The tumor tissues showed positive expression of high-molecular-weight cytokeratin (CK34βE12; **Figure 1(v)B**), cytokeratin 5/6 (CK5/6; **Figure 1(v)C**), glial fibrillary acidic protein (GFAP; **Figure 1(v)E**), Ki-67 protein (Ki-67; **Figure 1(v)F**), tumor protein p63 (p63; **Figure 1(v)G**), and smooth muscle actin (SMA; **Figure 1(v)H**), whereas CD10 (**Figure 1 (v)A**) and desmin (**Figure 1 (v)D**) were negative.

FISH analysis: A break-apart rearrangement of the *PLAG1* gene locus in the tumor cells, with a positivity rate of 86% (**Figure 1(vi)**).

Pathologic diagnosis: CXPA of the palate, the tumor is composed of a background of pleomorphic adenoma and the cancer component is mucoepidermoid carcinoma and solid-type adenoid cystic carcinoma.

### 2.2 Case 2

A 53-year-old female patient presented with a mass in the right parotid area for more than 2 years; the mass had enlarged painlessly over the previous 6 months, and she had not received prior intervention. Physical examination: irregular mass (about 5.0×5.0×3.0 cm) was palpated in the right postauricular and preauricular region, with poorly defined borders, medium texture, indistinct deep palpation, and no pressure pain. The right corner of the mouth showed a slight weakness in puffing the cheeks, no enlarged lymph nodes were palpable in the submandibular region bilaterally, and the secretion of the parotid duct orifices was normal. The preoperative clinical photograph showed visible swelling of the right parotid region with facial asymmetry compared with the contralateral side, while the postoperative photograph demonstrated the surgical incision in the right preauricular and postauricular regions (**Figure 2(i)**). Diagnostic imaging: The non-contrast CT scan showed a clearly demarcated soft tissue mass in the right parotid gland (**Figure 2(ii)**). No significant family history or specific allergies were documented. Detailed past medical, psychosocial, and genetic history was not available in the archival medical records.

**Figure 2:**
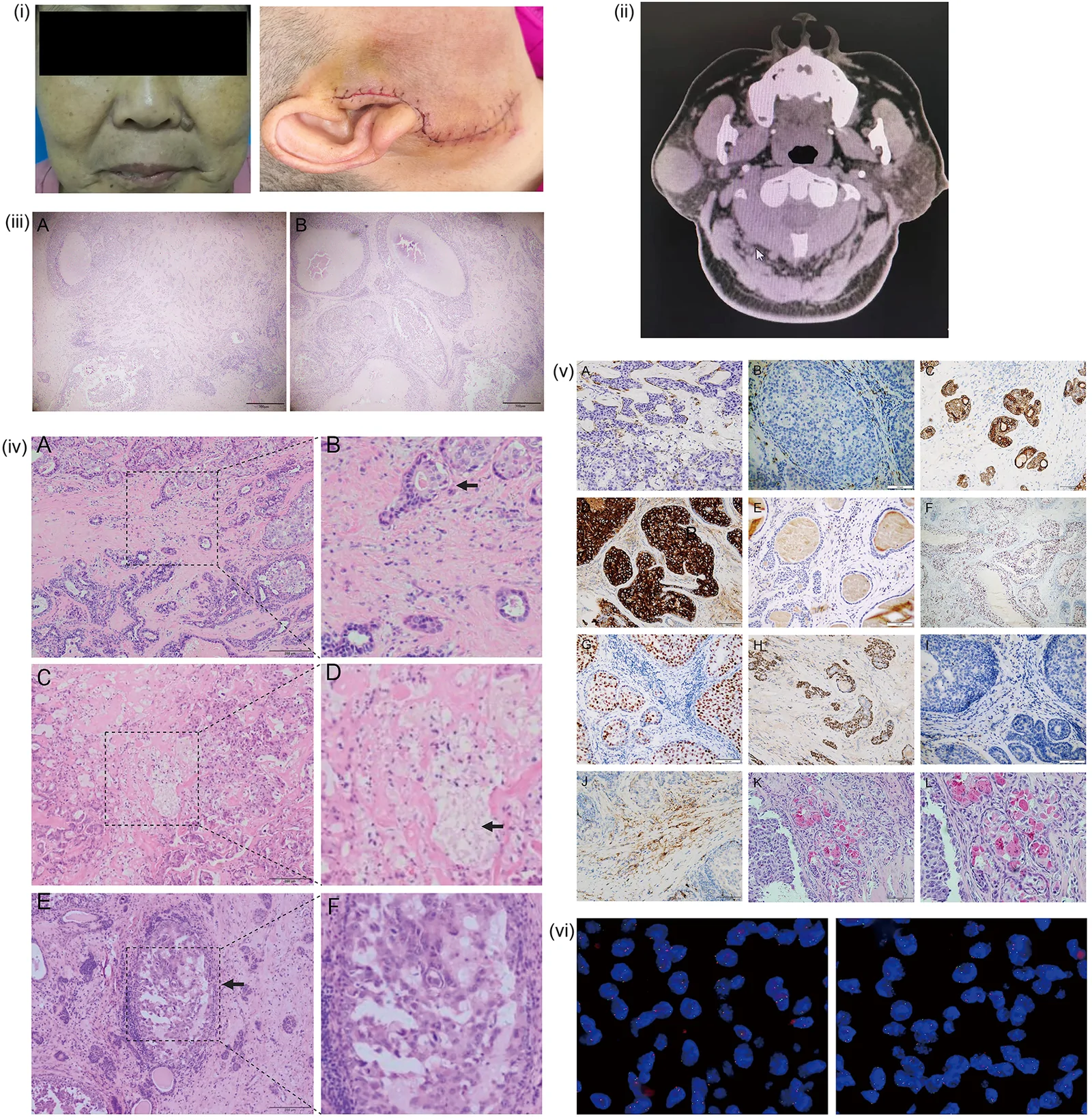
Clinical, radiological, histopathological, immunohistochemical, special staining, and molecular findings of case 2. **(i)** Clinical photographs showing swelling of the right parotid region with facial asymmetry preoperatively (left) and the surgical incision in the right preauricular and postauricular regions postoperatively (right). **(ii)** Axial radiographic image of Case 2. **(iii)** Representative low-power H&E images showing marked histological heterogeneity and the coexistence of morphologically distinct areas within the tumor **(A, B)**. **(iv)** Histopathological results of Case 2. **(A, B)** Bilayered duct-like structures containing homogeneous eosinophilic mucus within their lumina (black arrows). Myoepithelial components and myxoid stroma are also present. **(C, D)** Mucous cells, epidermoid cells, and mucin pools are visible (black arrows). **(E, F)** A “comedone-like” structure of necrosis is observed (black arrows). **(v)** Immunohistochemistry and special staining results of Case 2. **(A)** S-100(+), **(B)** CK7(+), **(C)** EMA(+), **(D)** CD117(-), **(E)** Calponin(+), **(F)** p63(+), **(G)** PR(-), **(H)** p53(+), **(I)** ER(+), **(J)** Ki-67(15% +). **(K, L)** Mucicarmine staining(+). **(vi)** FISH results of Case 2. Using break-apart probes targeting the *PLAG1* gene locus, the detection of split red and green signals indicates a rearrangement of the *PLAG1* gene. The fused yellow signals represent an intact gene locus.

#### 2.2.1 Preoperative diagnosis: parotid tumor, nature to be determined

Operative findings: On March 8, 2019, the patient underwent a right superficial parotidectomy with tumor excision. Intraoperatively, a firm, poorly demarcated mass measuring 4.0 × 4.0 × 3.0 cm was identified, which was closely adherent to branches of the facial nerve. The superficial lobe of the parotid gland along with the tumor was completely excised, and the specimen was submitted for routine pathological examination.

Pathological examination. Grossly, the tumor exhibited a nodular growth pattern and was mostly well-circumscribed. Microscopically, it displayed diverse cellular morphology and complex architecture. Representative low-power H&E sections demonstrated the coexistence of morphologically distinct areas within the tumor, with variable glandular, cribriform, and solid growth patterns (**Figures 2(iii)A, B**). At higher magnification, glandular epithelial and myoepithelial components were identified in association with myxochondroid stroma (**Figures 2(iv)A, B**). Mucous cells, epidermoid cells, and mucin pools were observed. A cribriform pattern, characterized by duct-like structures with intra-luminal projections, was present within glandular spaces (**Figures 2(iv)C, D**). Furthermore, the tumor cells were arranged in solid epithelial nests, with central necrosis in larger nests forming a “comedone-like” structure (**Figures 2(iv)E, F**).

Additional pathological parameters: The tumor measured approximately 5.0 × 5.0 × 3.0 cm clinically and 4.0 × 4.0 × 3.0 cm intraoperatively. Prominent comedone-like necrosis was present, and the Ki-67 proliferation index was approximately 15%, although no quantitative mitotic count was available. Detailed histological margin status, perineural and lymphovascular invasion, depth/extent of invasion, overall histological grade, and complete pathological stage were not reliably documented. No neck dissection was performed; therefore, pathological nodal status and extranodal extension could not be assessed.

Immunohistochemical results: The tumor tissues showed positive expression of S-100 protein (S-100; **Figure 2(v)A**), cytokeratin 7 (CK7; **Figure 2(v)B**), epithelial membrane antigen (EMA; **Figure 2(v)C**), p63 (**Figure 2(v)F**), Calponin (**Figure 2(v)E**), estrogen receptor (ER; **Figure 2(v)I**), and tumor protein p53 (p53; **Figure 2(v)H**); negative expression of CD117 (**Figure 2(v)D**), and progesterone receptor (PR; **Figure 2(v)G**); Ki-67 (15%, **Figure 2(v)J**);

Special staining results: Mucicarmine staining was positive in the tumor tissue (**Figures 2(v)K, L**).

FISH analysis: A break-apart rearrangement of the *PLAG1* gene locus in the tumor cells, with a positivity rate of 85% (**Figure 2(vi)**).

Pathologic diagnosis: CXPA of the right parotid, the tumor is composed of a background of pleomorphic adenoma and the cancer component is mucoepidermoid carcinoma and salivary gland ductal carcinoma.

Overall diagnostic assessment: In both cases, clinical examination and CT findings were nonspecific and insufficient for a definitive preoperative diagnosis. The differential diagnosis included pleomorphic adenoma and other benign or malignant salivary gland tumors. The main diagnostic challenge was marked intratumoral histological heterogeneity. Definitive diagnoses were established postoperatively by comprehensive histopathological examination, with immunohistochemistry, special staining, and PLAG1 FISH serving as supportive ancillary studies. The comparative diagnostic pathways of the two cases are summarized in **Figure 3**, and their clinical timelines are presented in **Table 1**.

**Figure 3:**
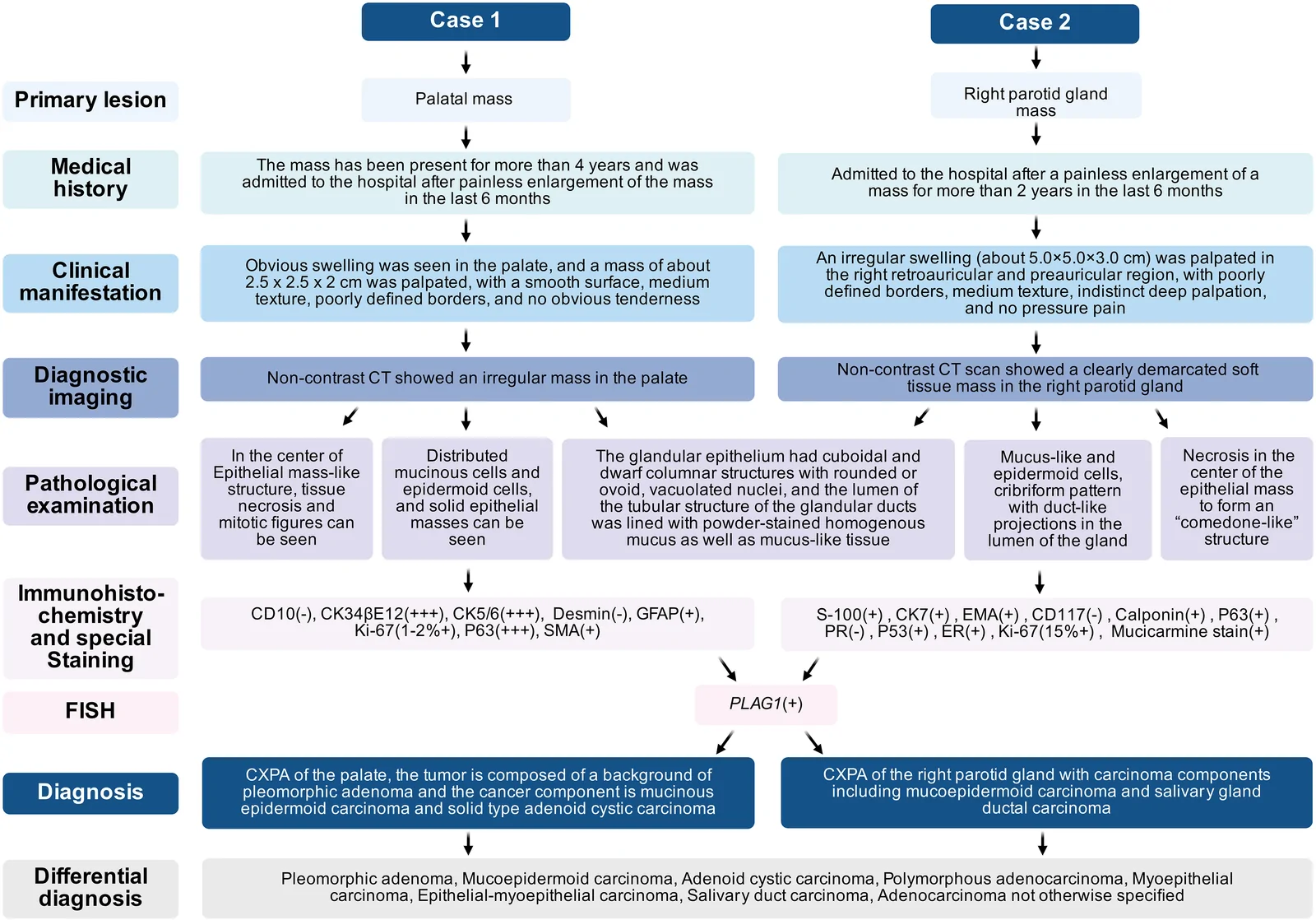
Diagnostic flowchart.

**Table 1:** Clinical timeline of the two cases.

Time/stage	Case 1: palatal CXPA	Case 2: right parotid CXPA
Initial disease course	Palatal mass present for approximately 4 years	Right parotid-region mass present for more than 2 years
6 months before presentation	Painless enlargement of the mass	Painless progressive enlargement of the mass
Main findings at presentation	Palatal mass measuring approximately 2.5 × 2.5 × 2.0 cm	Preauricular and postauricular mass measuring approximately 5.0 × 5.0 × 3.0 cm, with mild weakness of the right corner of the mouth
Preoperative imaging	CT showed an irregular palatal mass	CT showed a soft-tissue mass in the right parotid gland
Date of surgery	October 8, 2020	March 8, 2019
Surgical procedure	Excision of the palatal mass	Right superficial parotidectomy with tumor excision
Intraoperative tumor size	2.0 × 2.0 × 1.5 cm	4.0 × 4.0 × 3.0 cm
Final diagnosis	CXPA with mucoepidermoid carcinoma and solid-type adenoid cystic carcinoma components	CXPA with mucoepidermoid carcinoma and salivary duct carcinoma components
PLAG1 FISH	Rearrangement positivity rate: 86%	Rearrangement positivity rate: 85%
Postoperative adjuvant treatment	No radiotherapy, chemotherapy, or other systemic antitumor treatment	No radiotherapy, chemotherapy, or other systemic antitumor treatment
Postoperative complications	No major surgery-related complications were documented	No major surgery-related complications were documented
Follow-up	3 years; no local recurrence or distant metastasis	2 years; no local recurrence or distant metastasis

Treatment and follow-up: Case 1 underwent excision of the palatal mass on October 8, 2020, and Case 2 underwent right superficial parotidectomy with tumor excision on March 8, 2019. Both tumors were grossly completely excised, although detailed histological margin status was unavailable. Neither patient underwent neck dissection or received postoperative radiotherapy, chemotherapy, or other systemic treatment. The rationale for these treatment decisions was not documented in the historical records. No major surgery-related complications, local recurrence, or distant metastasis were identified during the available follow-up periods of 3 years for Case 1 and 2 years for Case 2. Detailed follow-up imaging, laboratory findings, and structured patient-reported outcomes were not systematically documented.

Patient perspective: During follow-up visits, both patients verbally expressed satisfaction with the treatment outcomes. However, structured patient-reported outcomes were not systematically documented in the original medical records.

## 3 Discussion

CXPA is an epithelial malignancy arising in primary or recurrent PA, also termed mixed tumor carcinoma or malignant mixed tumor. Pathologically, it shows benign PA features coexisting with malignant epithelial components (9). It is rare: 50–80% occur in parotid, 10–15% in submandibular, and the rest in minor salivary glands. Clinically, it often presents as rapid enlargement of a longstanding mass (>3 years), with pain, facial palsy, or nerve symptoms; metastases commonly to bone, lung, lymph nodes, and liver (5, 6). Diagnosis is challenging due to nonspecificity, requiring pathology, immunohistochemistry, and FISH. We report two cases with mucoepidermoid, adenoid cystic, and salivary ductal carcinoma components, rare and diagnostically complex.

### 3.1 Pathogenesis

CXPA arises from malignant transformation of pleomorphic adenoma. Risk factors include long history, large size, hyalinization, age, smoking, and radiotherapy (1, 3, 5, 6, 10, 11); size>2cm raises suspicion (4). Pathogenesis remains unclear. Our two cases had dual components: mucoepidermoid, adenoid cystic, and salivary duct carcinomas, reflecting lineage diversity. Heterogeneity suggests microenvironment involvement in proliferation, invasion, migration, and drug resistance. Mucoepidermoid carcinoma links to chronic inflammation (12), driving proliferation and DNA damage. Thus, CXPA likely results from multiple factors.

### 3.2 Diagnostic imaging

Imaging (ultrasound, CT, MRI) is valuable in CXPA evaluation, assessing tumor size, shape, relationship with surrounding tissues, and margin clarity and internal homogeneity to suggest benign or malignant nature. In our study, non-contrast CT revealed an irregular palatal mass in Case 1 and a well-defined right parotid soft tissue mass in Case 2. CXPA on CT typically presents as lobular, irregular, unevenly enhanced masses with smooth margins, liquefaction necrosis, cystic degeneration, and coarse or mixed calcification (13). MRI often shows unclear borders, irregular morphology, absent or incomplete capsule, peripheral infiltration, and low signal with heterogeneous enhancement on T1WI and T2WI (14). Fine-needle aspiration cytology and immunostaining may support diagnosis. However, given CXPA’s complex structure, imaging alone cannot confirm diagnosis; it must be combined with clinical findings, history, histopathology, and immunohistochemistry (15–17).

### 3.3 Histopathologic morphology

Pathological examination is critical for CXPA diagnosis and grading. Meticulous histologic analysis is indispensable (17). The evaluation determines tumor type and grade, guiding therapy. The hallmark pathological feature is the coexistence of benign PA and malignant components under light microscopy; the malignant cells show nuclear atypia, mitotic figures, and increased nuclear-to-cytoplasmic ratio with hyperchromatic nuclei. The specific histopathological phenotype varies depending on the nature of the malignant element present within the tumor (2). In Case 1, homogeneous eosinophilic mucus was observed within the lumina of gland-like structures, with myxoid tissue distributed throughout the tumor (**Figures 1(iv)A, B**). The tumor also exhibited sheet-like arrangements of mucous cells and epidermoid cells, alongside solid epithelial nests (**Figures 1(iv)C, D**). Some cells formed large epithelial masses showing central necrosis, with identifiable mitotic figures (**Figures 1(iv)E, F**). In Case 2, histopathological examination revealed components of pleomorphic adenoma, including glandular epithelium, myoepithelial structures, and myxochondroid areas (**Figures 2(iv)A, B**). The malignant component was characterized by prominent mucous cells, epidermoid cells, mucin pools, and the presence of a typical cribriform pattern (**Figures 2(iv)C, D**) as well as “comedone-like” structure(**Figures 2(iv)E, F**).

In the present cases, the MEC component was diagnosed primarily on the basis of characteristic morphology, including the coexistence of mucous and epidermoid cells with mucin production. In Case 1, CK5/6, CK34βE12, and p63 expression provided supportive evidence of squamoid/basal differentiation. In Case 2, mucicarmine staining confirmed mucin production, while CK7 and p63 supported glandular and epidermoid/basal differentiation, respectively. These ancillary findings were interpreted as supportive rather than specific. PLAG1 rearrangement was considered evidence of the underlying pleomorphic adenoma/CXPA lineage rather than a molecular marker of MEC itself. The solid-type adenoid cystic carcinoma component in Case 1 and the salivary duct carcinoma component in Case 2 were likewise identified primarily based on their characteristic histomorphological features, with the available immunohistochemical findings interpreted as supportive rather than independently diagnostic.

### 3.4 Histologic origin

Salivary glands have luminal (acinar and ductal) and abluminal (myoepithelial and basal) cells, which can re-enter cell cycle for neoplastic transformation. Tumors contain glandular epithelial cells and stromal fibroblasts interacting in the microenvironment. Mucoepidermoid carcinoma comprises mucous, intermediate (possibly modified myoepithelial), and epidermoid cells, originating from salivary duct reserve or myoepithelial cells (18). Adenoid cystic carcinoma is basaloid with epithelial and myoepithelial cells in tubular, cribriform, and solid patterns, arising from intercalated duct reserve or basal cells (19). Salivary duct carcinoma consists of cuboidal or columnar ductal cells derived from excretory duct reserve cells (20). In our two CXPA cases, malignant cells showed enlarged hyperchromatic nuclei, prominent nucleoli, and frequent mitoses. CXPA mainly arises from malignant transformation of pleomorphic adenoma (21), sharing origin; epithelial-mesenchymal transition and myoepithelial cells play key roles (2).

### 3.5 Immunohistochemistry

Immunohistochemistry is essential for CXPA diagnosis, providing tumor typing and biological behavior information. In Case 1, CD10 negativity in carcinomatous areas indicated phenotype loss after malignant transformation and supported carcinoma diagnosis (22). CK34βE12 strong positivity indicated a basal-cell-like phenotype, which is typically associated with aggressive tumor behavior. GFAP positivity indicated the presence of myoepithelial cells and neurogenic features within the tumor, aiding identification of an adenoid cystic carcinoma component. SMA positivity indicated myoepithelium-derived elements in the tumor tissue, whereas Desmin negativity supported the existence of non-myoepithelial components (23, 24). Ki-67 >5% indicated active tumor cell proliferation. In Case 2, S-100 positivity was observed in myxoid and chondroid stroma and in tumor cells (25). Myoepithelial markers (CK5/6, CK7, S°100, SMA, GFAP, p63, Calponin) and glandular markers (CK7, EMA) were evaluated. p63 and Calponin positivity indicated myoepithelial cells. CD117 negativity indicated malignant transformation of ductal epithelial cells and supported the presence of a salivary duct carcinoma component. p53 nuclear positivity indicated malignancy, consistent with CXPA (26). Ki-67 >5% indicated active proliferation. ER positivity combined with PR negativity within ductal structures excluded metastatic breast carcinoma. Mucicarmine positivity indicated mucoid cells in the tumor tissue.

### 3.6 Fluorescence *in situ* hybridization analysis

FISH provided molecular evidence supporting the diagnosis; gene rearrangement (PLAG1) is a hallmark genetic alteration. Both cases showed PLAG1 break-apart rearrangements, specific for PA and CXPA (27). Combined with imaging, pathology, and IHC, FISH confirmed CXPA in both cases.

### 3.7 Differential diagnosis

CXPA is characterized by the coexistence of both benign pleomorphic adenoma components and diverse malignant elements. Therefore, accurate diagnosis requires careful identification of both the benign and carcinomatous areas, combined with thorough clinical history assessment and extensive pathological sampling. In the present cases, the principal diagnostic challenge was the marked intratumoral heterogeneity and the coexistence of two morphologically distinct malignant components. Accordingly, the differential diagnosis was based primarily on histomorphology, while immunohistochemistry, special staining, and PLAG1 FISH were interpreted as supportive ancillary findings. The differential diagnosis of CXPA primarily involves distinguishing it from other types of salivary gland tumors and non-neoplastic lesions. In the two cases presented in this report, differentiation is necessary not only from pleomorphic adenoma, but also from other entities such as mucoepidermoid carcinoma, adenoid cystic carcinoma, polymorphous low-grade adenocarcinoma, myoepithelial carcinoma, epithelial-myoepithelial carcinoma, salivary duct carcinoma, and adenocarcinoma not otherwise specified, based on their respective clinicopathological features.

#### 3.7.1 Pleomorphic adenoma

PA is benign without malignant components. Our two cases contained unequivocal malignant areas with positive carcinoma-associated markers, thus excluding PA (28).

#### 3.7.2 Mucoepidermoid carcinoma

Mucoepidermoid carcinoma is characterized by mucous, epidermoid, and intermediate cells with mucin production. Immunohistochemistry, mucicarmine staining, and MAML2 rearrangement may provide supportive evidence (29–31). In the present cases, MEC was diagnosed primarily based on morphology, while the pleomorphic adenoma background and additional malignant components supported CXPA rather than pure MEC.

#### 3.7.3 Adenoid cystic carcinoma

AdCC has bidirectional epithelial/myoepithelial differentiation, with cribriform/tubular/solid patterns, positive for S-100, MYC, P63, CK7, Calponin, and CD117 (32, 33). Our cases additionally contained PA areas (myoepithelial, myxoid) and MEC features, plus PLAG1 positivity, excluding pure AdCC (26).

#### 3.7.4 Polymorphous low-grade adenocarcinoma

PLGA consists of myoepithelial and ductal cells with lobular, cribriform, and tubular patterns, positive for actin, S-100, vimentin, EMA, and CK; it lacks distinct dual populations and typically lacks PLAG1 rearrangement (27, 34). Our cases had both ductal and myoepithelial components and PLAG1 positivity, excluding PLGA.

#### 3.7.5 Myoepithelial carcinoma

MyoCa often arises from PA, shows trabecular/epithelioid cells, positive for CK°AE1/AE3 and Calponin; negative for CK7, CK5/6, p63, S°100, SMA, AB, and PAS (8, 35, 36). Our cases were positive for CK7, CK5/6, P63, S-100, and PLAG1, excluding MyoCa (27).

#### 3.7.6 Epithelial-myoepithelial carcinoma

EMC has biphasic ducts (inner ductal, outer clear myoepithelial), positive for myoepithelial markers (CD10, p63, Calponin, CK5/6, S°100) and ductal markers (CAM5.2, CK7, EMA), negative for Desmin and PAS, and usually PLAG1-negative (37, 38). Our cases showed discordant architecture and PLAG1 positivity, excluding EMC.

#### 3.7.7 Salivary duct carcinoma

SDC shows intraductal proliferations with cribriform/papillary/solid nests and comedone-like necrosis, positive for CK7, CEA, EMA, GCDFP-15, AR, negative for ER/PR, high Ki-67, and typically PLAG1-negative (27, 39–41). Our cases had heterogeneous histology (myxochondroid, mucous cells) and PLAG1 positivity, excluding pure SDC.

#### 3.7.8 Adenocarcinoma not otherwise specified

NOS shows unclassifiable glandular differentiation, positive for CK7, p16, HBME-1, CAM5.2, negative for EMA and CK5/6 (8, 42). Our cases were positive for both EMA and CK5/6, excluding NOS.

### 3.8 Clinical implications

The recognition of dual malignant components in carcinoma ex pleomorphic adenoma (CXPA) has important clinical implications for diagnosis, pathological evaluation, and subsequent management. Because different malignant components may coexist within the same tumor, a limited preoperative biopsy or fine°needle aspiration may sample only one component and thereby underestimate the histological complexity of the lesion. Accordingly, extensive sampling of the resection specimen and careful examination of morphologically distinct areas are important to avoid incomplete classification. Immunohistochemistry and special stains may provide supportive evidence for the characterization of individual malignant components, whereas molecular testing such as PLAG1 FISH may support an underlying pleomorphic adenoma/CXPA lineage in the appropriate morphological context (43).

The biological behavior of CXPA may also be influenced by the histological subtype of the malignant component together with other established pathological risk features. For example, adenoid cystic carcinoma is associated with a propensity for perineural invasion and distant metastasis (44), whereas salivary duct carcinoma is characterized by aggressive local behavior and a relatively high rate of regional lymph-node metastasis (45). Therefore, identification and documentation of each malignant component, together with the assessment of the extent of invasion, surgical margins, perineural invasion, lymphovascular invasion, and nodal status, may provide clinically relevant information for postoperative risk stratification, multidisciplinary treatment planning, and follow-up. Our cases highlight the importance of comprehensive pathological assessment rather than relying solely on the dominant or initially sampled malignant component.

### 3.9 Treatment and prognosis

Surgical resection is the primary treatment, and the approach depends on tumor location, extent, and stage. Adjuvant radiotherapy or chemotherapy may be added when indicated. Lymphadenectomy is performed based on nodal metastasis status (21). In the present cases, Case 1 underwent local excision of the palatal tumor, whereas Case 2 underwent right superficial parotidectomy with tumor excision. Neither patient underwent neck dissection. Postoperatively, neither patient received radiotherapy, chemotherapy, or other systemic antitumor treatment, and no major surgery-related complications were documented. The specific clinical rationale for not performing neck dissection or postoperative adjuvant therapy was not recorded in the available historical medical records and was therefore not retrospectively inferred.

Early-stage tumors with clear margins generally yield good outcomes, whereas aggressive or metastatic lesions require wider excision combined with postoperative radiotherapy. Prognosis is influenced by pathological grade, site, size, and neural or osseous invasion, with depth of invasion being the most critical factor. Specifically, invasion <5 mm beyond the capsule with clear margins predicts a favorable prognosis (3, 11); conversely, advanced stage, higher T stage, nodal metastasis, high histological grade, and deeper invasion are associated with poorer outcomes (46). In the present cases, no local recurrence or distant metastasis was identified during the available follow-up periods of 3 years for Case 1 and 2 years for Case 2. However, these relatively limited follow-up periods should be considered when interpreting the outcomes.

Therefore, surgical resection remains the cornerstone, but treatment strategies should integrate histopathological and immunohistochemical findings. Close postoperative follow-up is essential for early detection. Because these were retrospective cases, several detailed pathological parameters, including precise histological margin information, quantitative depth or extent of invasion, and complete pathological staging, could not be reliably retrieved from the original records and were not retrospectively inferred.

## 4 Conclusion

A review of the literature shows that most CXPA cases harbor a single malignant component, whereas those with two distinct components are rare. In our series, Case 1 had MEC combined with AdCC, while Case 2 had MEC combined with SDC. These different components may be associated with distinct biological behaviors: AdCC exhibits a high propensity for perineural invasion and distant metastasis, whereas SDC displays marked local aggressiveness, high proliferative activity, and lymphatic spread. MEC is often low-grade, but both AdCC and SDC are high-grade malignancies. Identification of the specific malignant components may provide clinically relevant information for pathological assessment, risk stratification, and subsequent management. Clinical history and symptoms offer limited diagnostic value; definitive diagnosis requires integration of imaging, histopathology, immunohistochemistry, and special stains. Moreover, PLAG1 FISH may provide additional evidence supporting the underlying PA/CXPA lineage (47–49). Our two cases were confirmed through this multimodal approach. Early detection and vigilant surveillance remain crucial for optimal management.

## Data Availability

The original contributions presented in the study are included in the article/supplementary material. Further inquiries can be directed to the corresponding authors.
